# A-Type Cranberry Proanthocyanidins Inhibit the RANKL-Dependent Differentiation and Function of Human Osteoclasts

**DOI:** 10.3390/molecules16032365

**Published:** 2011-03-11

**Authors:** Shinichi Tanabe, Juliana Santos, Vu Dang La, Amy B. Howell, Daniel Grenier

**Affiliations:** 1 Groupe de Recherche en Écologie Buccale, Faculté de Médecine Dentaire, Université Laval, 2420 Rue de La Terrasse, Quebec City, QC, G1V0A6, Canada; E-Mails: stanabequebec@yahoo.co.jp (S.T.); jusantosbh@hotmail.com (J.S.); danglv2001@yahoo.com (V.D.L.); 2 Marucci Center for Blueberry and Cranberry Research, Rutgers, The State University of New Jersey, Chatsworth, NJ 08019, USA; E-Mail: ahowell@aesop.rutgers.edu

**Keywords:** proanthocyanidin, cranberry, periodontitis, bone resorption, osteoclast

## Abstract

This study investigated the effect of A-type cranberry proanthocyanidins (AC-PACs) on osteoclast formation and bone resorption activity. The differentiation of human pre-osteoclastic cells was assessed by tartrate-resistant acid phosphatase (TRAP) staining, while the secretion of interleukin-8 (IL-8) and matrix metalloproteinases (MMPs) was measured by ELISA. Bone resorption activity was investigated by using a human bone plate coupled with an immunoassay that detected the release of collagen helical peptides. AC-PACs up to 100 µg/mL were atoxic for osteoclastic cells. TRAP staining evidenced a dose-dependent inhibition of osteoclastogenesis. More specifically, AC-PACs at 50 µg/mL caused a 95% inhibition of RANKL-dependent osteoclast differentiation. This concentration of AC-PACs also significantly increased the secretion of IL-8 (6-fold) and inhibited the secretion of both MMP-2 and MMP-9. Lastly, AC-PACs (10, 25, 50 and 100 µg/ml) affected bone degradation mediated by mature osteoclasts by significantly decreasing the release of collagen helical peptides. This study suggests that AC-PACs can interfere with osteoclastic cell maturation and physiology as well as prevent bone resorption. These compounds may be considered as therapeutic agents for the prevention and treatment of periodontitis.

## 1. Introduction

The cranberry (*Vaccinium macrocarpon*), a native North American fruit, has been widely investigated for its diverse beneficial effects for human health, primarily those related to its anti-adherence activity [1]. Indeed, cranberry proanthocyanidins, which present a unique oligomeric structure with A-linkage that differs them from B-type proanthocyanidins found in other berry fruits, have been intensively investigated. More specifically, cranberry A-type proanthocyanidins (AC-PACs) demonstrated anti-adhesion effect against *Escherichia coli*, whereas B-type proanthocyanidins from other fruits were devoid of anti-adhesion properties [2,3]. Proanthocyanidin-enriched cranberry extracts have also presented a variety of potential benefits for oral health, such as inhibition of biofilm formation and acid production by cariogenic bacteria [4] as well as modulation of the inflammatory response to periodontopathogens [5] and inactivation of bacteria-related proteolytic enzymes [6]. Additionally, our laboratory showed that AC-PACs were able to inhibit matrix metalloproteinase (MMP) production by human macrophages stimulated with *Aggregatibacter actinomycetemcomitans* lipopolysaccharide (LPS), as well as to reduce MMP-1 and -9 catalytic activities [7]. We have also demonstrated that AC-PACs efficiently neutralized *Porphyromonas gingivalis* virulence properties and modulated the inflammatory response of epithelial cells to this periodontopathogen [8].

The resorption of alveolar bone is a typical hallmark of periodontal disease, a multifactorial disorder triggered by the accumulation of specific bacterial species organized in a biofilm and present in subgingival sites. These periodontopathogens, mostly Gram-negative and strictly anaerobic, are able to stimulate a host immune response, which in turn leads to a destructive inflammatory process [9]. The secretion of proinflammatory mediators, including cytokines, chemokines and prostaglandins, allow the propagation of inflammation within gingival tissues and the expansion of the process to the adjacent alveolar bone [10].

Alveolar bone destruction is mediated by the recruitment and differentiation of osteoclasts into their mature phenotype. These cells derive from hematopoietic monocyte/macrophage precursors under the action of receptor activator of nuclear factor kappa-B ligand (RANKL) and macrophage colony-stimulating factor (M-CSF). Once activated, resorptive osteoclasts attach to the bone surface and promote mineral dissolution by acidification of the sub-osteoclastic microenvironment [11]. Subsequently, the demineralized organic matrix of bone is degraded by secreted proteases such as cathepsin K and MMPs [11]. It has been demonstrated that osteoclastogenesis is enhanced during periodontal disease due to the accumulation of inflammatory cytokines, which will either stimulate osteoclast proliferation or promote the differentiation and maturation of progenitor cells [12,13]. Accordingly, the modulation of osteoclast formation and function is pointed as one of the therapeutic targets in the prevention of alveolar bone loss associated with periodontal disease.

Since AC-PACs present a number of biological activities that might be relevant to the control of tissue destruction occurring in periodontal disease, we hypothesized that these natural compounds can also interfere with bone resorption mediated by osteoclasts. Therefore, the aim of the present study was to investigate the effect of AC-PACs on osteoclast differentiation and physiology, as well as on its bone-resorbing activity.

## 2. Results and Discussion

### 2.1. A-type cranberry proanthocyanidins

**Figure 1:**
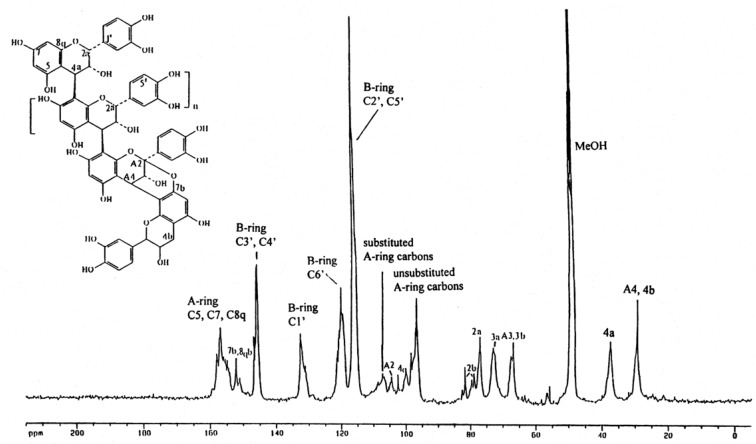
^13^C-NMR spectrum of cranberry proanthocyanidins showing the presence of A-type linkages.

Characterization of the AC-PACs fraction was made by ^13^C-NMR. As shown in Figure 1, the proanthocyanidin molecules consist of epicatechin units presenting mainly a degree of polymerization (DP) of 4 and 5 and containing at least one A-type linkage, as previously reported [14].

### 2.2. Cytotoxicity

**Figure 2:**
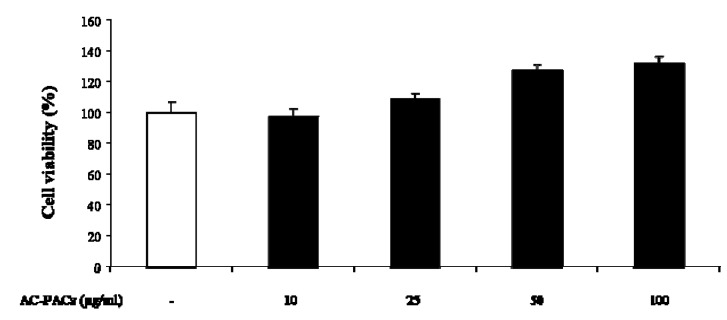
Cytotoxic effect of AC-PACs on osteoclastic cells as measured by the MTT assay.

As reported in Figure 2, AC-PACs did not exhibit any detrimental effect on cell viability at concentrations ranging from 10 to 100 µg/mL. 

Conversely, a cell proliferation increase of up to 32 ± 5% was observed at the highest concentrations tested, indicating the absence of any significant toxic effects towards osteoclasts.

### 2.3. Osteoclast formation

**Figure 3:**
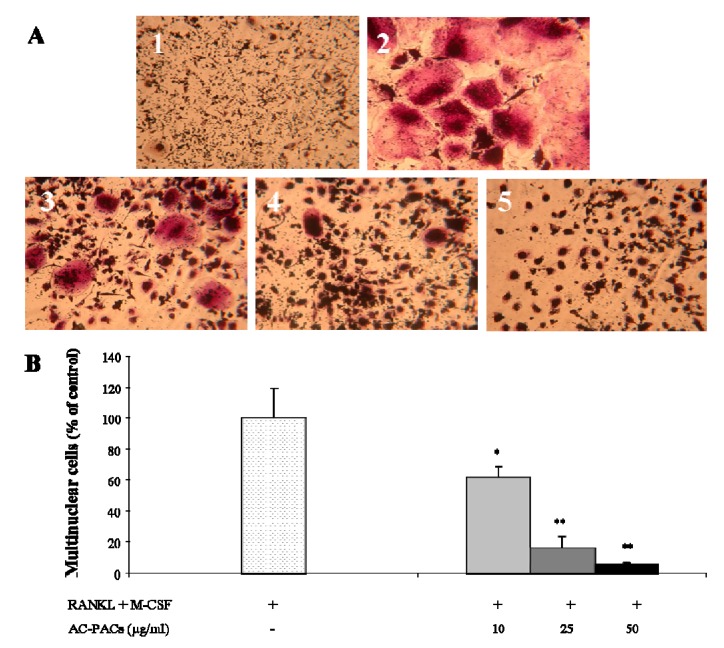
Inhibitory effect of AC-PACs on the differentiation of human pre-osteoclasts. Cells were treated with various concentrations of AC-PACs and cultivated in the presence of both M-CSF and RANKL. **(A)** TRAP staining was performed to evidence multinuclear cells. 1) Cells treated with M-CSF only (negative control); 2) Cells treated with M-CSF + RANKL (positive control); 3) Cells treated with M-CSF + RANKL + 10 µg/mL AC-PACs; 4) Cells treated with M-CSF + RANKL + 25 µg/mL AC-PACs; 5) Cells treated with M-CSF + RANKL + 50 µg/mL AC-PACs. **(B)** % of multinuclear TRAP-stained cells: Control – 100%; 10µg/mLAC-PAC - 62 ± 7%; 25µg/mL AC-PAC – 16± 7%; 50µg/mL AC-PAC – 5 ± 1%. * *p* < 0.05, ** *p* < 0.01.

The degree of osteoclast formation was evaluated by quantification of TRAP-positive stained multinucleated cells. Within the range of concentrations tested (10–50 µg/mL), AC-PACs were able to decrease the formation of differentiated osteoclasts (TRAP-positive multinucleated cells) in a dose-dependent manner (Figure 3A). A significant inhibition (p < 0.05) of osteoclast differentiation could be observed, even when cells were treated with the lowest concentration of AC-PACs (10 µg/mL) (Figure 3B). More specifically, AC-PACs at final concentrations of 10, 25 and 50 µg/mL caused an inhibition on cell maturation of 38 ± 7%, 84 ± 7%, and 95 ± 1%, respectively (Figure 3B). The impairment of the maturation process of pre-osteoclastic cells after being exposed to both RANKL and M-CSF suggests that AC-PACs may hamper osteoclast formation.

### 2.4. Interleukin and matrix metalloproteinase secretion

**Figure 4:**
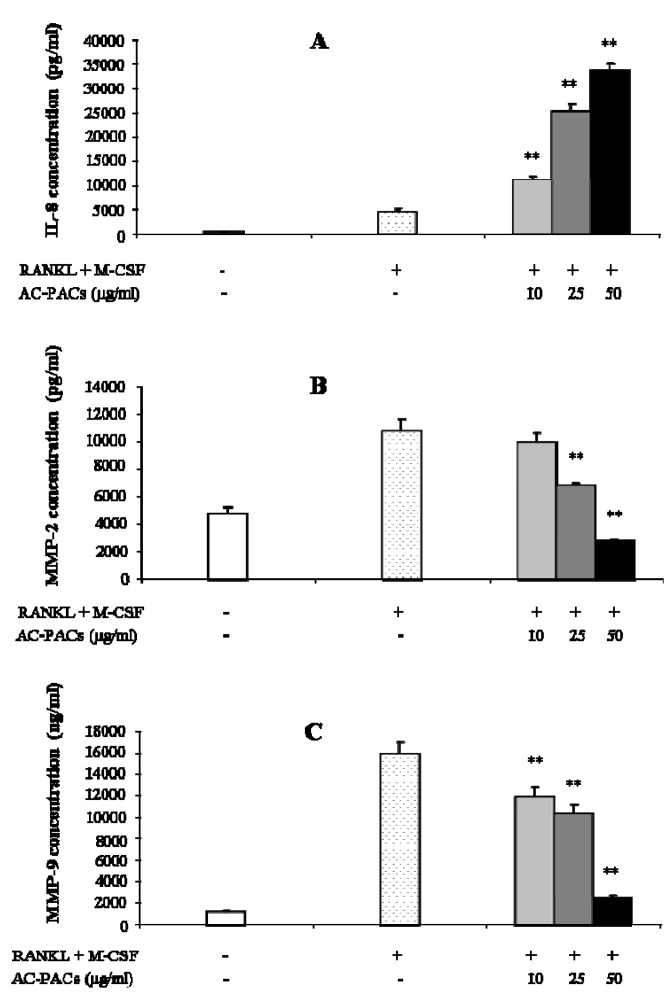
Effect of AC-PACs on secretion of IL-8 and MMPs by osteoclastic cells treated with AC-PACS. **(A)** IL-8 secretion. **(B)** MMP-2 secretion. **(C)** MMP-9 secretion. ** *p* < 0.01.

The effect of AC-PACs on the secretion of IL-8, a mediator involved in osteoclastogenesis, and MMP-2 and -9, proteinases related to the resorptive activity of osteoclasts, is reported in Figure 4. IL-8 secretion was significantly increased by AC-PACs at 10, 25 and 50 µg/mL by 2-, 6- and 7-fold, respectively (Figure 4A). Conversely, treatment of osteoclast cells with AC-PACs at 25 and 50 µg/mL decreased the levels of MMP-2 production by 36 ± 2% and 75 ± 1%, respectively (Figure 4B). MMP-9 secretion was also significantly reduced by AC-PACs treatment at 10, 25 and 50 µg/mL (Figure 4C), showing inhibition levels of 25 ± 6, 35 ± 5 and 84 ± 1%, respectively. 

Since the production of IL-8 by osteoclasts has been previously indicated as one of the steps of the signalling pathway for the recruitment of cells involved in bone remodelling [15], the demonstrated effect of AC-PACs on the secretion of this cytokine confirms the potential of these molecules to play a role in the regulation mechanisms of bone destruction. Gelatinases (MMP-2 and -9) derived from both resident and inflammatory cells participate in the pathological destruction of connective tissue. Accordingly, the important decrease of the secretion of both proteinases can be a quite valuable outcome regarding the control of connective tissue destruction mediated by such host-derived enzymes in the context of periodontal disease. Indeed, the AC-PACs were already shown to affect MMP-1 and -9 secretion by LPS-stimulated macrophages, which was attributed to a reduction on the phosphorylation of five intracellular kinases and the inhibition of nuclear factor-kappa B (NF-κB) [7]. However, the exact mechanism by which AC-PACs inhibit MMP secretion still deserves further investigation.

### 2.5. Bone resorption

**Figure 5:**
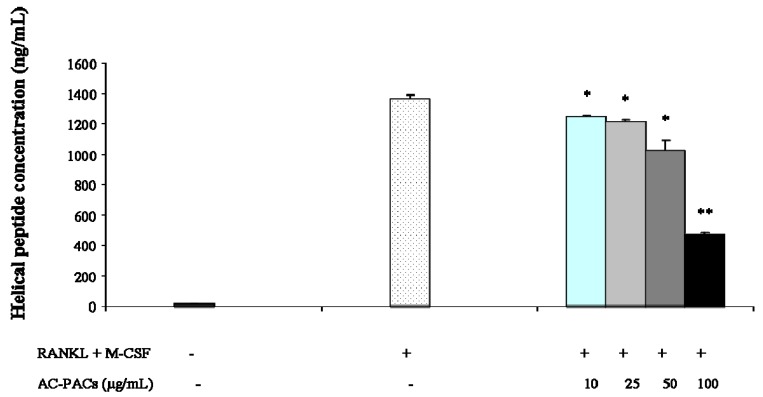
Effect of AC-PACs on bone resorption mediated by osteoclasts. * *p* < 0.05, ** *p* < 0.01.

The quantification of collagen helical peptides was performed by an enzymatic immunoassay to investigate the effect of AC-PACs on bone resorption activity of osteoclasts. A significant decrease (p < 0.05) on the release of helical peptides from the bone matrix was observed when osteoclastic cells were treated with any of the tested concentrations of AC-PACs (Figure 5). More specifically, a 66 ± 1% inhibition was obtained with AC-PACs at 100 µg/mL. This result further confirms the inhibitory effect of AC-PACs on osteoclastic activity.

## 3. Experimental

### 3.1. General

The MTT [3-(4,5-diethylthiazol-2-yl)-2,5-diphenyltetrazolium diphenyltetrazolium bromide] cell proliferation assay kit was purchased from Roche Diagnostics (Mannheim, Germany). The Human Poietics® osteoclast precursor cell system and OsteoAssay^TM^ human bone plate were obtained from Lonza Inc. (Allendale, NJ, USA). The MicroVue helical peptide enzyme immunoassay kit was purchased from Quidel Corp. (San Diego, CA, USA), while the interleukin-8 (IL-8), MMP-2 and MMP-9 ELISA kits were obtained from R&D Systems (Minneapolis, MN, USA). The Leukocyte Acid Phosphatase kit was supplied by Sigma Chemical Co. (St. Louis, MO, USA).

### 3.2. Preparation of A-type cranberry proanthocyanidins

Cranberry proanthocyanidins were isolated from cranberry fruit (*Vaccinium macrocarpon* Ait.) using solid-phase chromatography according to a well-established method for proanthocyanidin isolation [3]. Briefly, cranberry fruit was homogenized with 70% aqueous acetone, filtered and the pulp discarded. The collected extract was concentrated under reduced pressure to remove acetone. The cranberry extract was suspended in water, applied to a preconditioned C_18_ solid phase chromatography column and washed with water to remove sugars, followed by acidified aqueous methanol to remove acids. The fats and waxes retained on the C_18_ sorbent were discarded. The polyphenolic fraction containing anthocyanins, flavonol glycosides and proanthocyanidins (confirmed using reverse phase HPLC with diode array detection) was eluted with 100% methanol and dried under reduced pressure. This fraction was suspended in 50% EtOH, applied to a pre-conditioned Sephadex LH-20 column which was washed with 50% EtOH to remove low molecular weight anthocyanins and flavonol glycosides. Proanthocyanidins adsorbed to Sephadex LH-20 were eluted from the column with 70% aqueous acetone, and monitored using diode array detection at 280 nm. The absence of absorption at 360 nm and 450 nm confirmed that anthocyanins and flavonol glycosides were removed. Acetone was removed under reduced pressure and the resulting purified proanthocyanidin extract freeze-dried. Methods including ^13^C-NMR, electrospray mass spectrometry, matrix-assisted laser desorption/ ionization time-of-flight mass spectrometry and acid catalyzed degradation with phloroglucinol have all been utilized to confirm the presence of A-type linkages and concentration of proanthocyanidins present in the extract [2,3,14].

### 3.3. Cytotoxicity

The cytotoxicity of AC-PACs was measured by the MTT assay according to the manufacturer’s protocol. Cells were seeded in 96-well culture plates and treated with AC-PACs at 10, 25, 50 and 100 µg/mL for two days. The cells were then incubated with MTT for 4 h. This allowed the formation of formazan crystals, which were solubilised overnight at 37 °C and the optical absorbance was measured on a microplate reader (Model-680, Bio-Rad Laboratories, Mississauga, ON, Canada) at a wavelength of 550 nm and a reference wavelength of 650 nm. Cytotoxicity was quantified as the relative decrease in the absorbance compared with untreated control cells.

### 3.4. Osteoclast formation

The human osteoclast precursor cells used in this study originated from haematopoietic stem cells isolated from the human bone marrow. Cell culture was initiated according to the manufacturer’s instructions. Upon thawing, cells were washed, suspended in Osteoclast Precursor Basal Medium (Lonza Inc.) containing 10% heat-inactivated fetal bovine serum, 2 mM L-glutamine, 100 units/mL of penicillin, 100 µg/mL of streptomycin, and supplemented with RANKL (66 ng/mL) and M-CSF (33 ng/mL). Cells were then seeded in 96-well plates (1 × 10^3^ cells/well in 200 µL). AC-PACs, prepared in sterile distilled water, were added to the cells at final concentrations of 10, 25 or 50 µg/mL. Cells incubated in the absence of AC-PACs were used as positive controls and cells incubated without RANKL served as negative controls since no differentiation was expected. Culture microplates were incubated for six days in a humidified atmosphere containing 95% air and 5% CO_2_ at 37 °C to allow differentiation of precursors into mature osteoclasts. As recommended by the manufacturer, no replacement of culture medium was performed during the incubation period. Mature osteoclast formation was estimated by staining cells with a Leukocyte Acid Phosphatase assay kit. Tartrate-resistant acid phosphatase (TRAP)-positive multinucleated cells were stained in dark-red and the number of stained cells was determined under the microscope (100× magnification). Duplicate counts of triplicate wells for each condition were performed, and the means ± standard deviations (SD) were calculated.

### 3.5. Interleukin and matrix metalloproteinase secretion

Culture supernatants from osteoclast precursor cells treated with various concentrations of AC-PACs for six days were collected and analyzed for IL-8, MMP-2 and MMP-9 production by means of commercial ELISA kits. The absorbance was read using a microplate reader at 450 nm with the wavelength correction set at 550 nm. The rated sensitivities of the commercial ELISA kits were 31.2 pg/mL for IL-8, 47 pg/mL for MMP-2 and 310 pg/ml for MMP-9.

### 3.6. Bone resorption

Osteoclast precursor cells were differentiated in OsteoAssay^TM^ human bone plates by the addition of RANKL (66 ng/mL) and M-CSF (33 ng/mL) followed by a four-day incubation. Thereafter, the culture medium was replaced by fresh medium containing AC-PACs at final concentrations of 10, 25, 50 or 100 µg/mL, and cells were further incubated for four days. Fresh medium without AC-PACs (but containing RANKL and M-CSF) was used as positive control (differentiated cells) and medium without RANKL (with M-CSF) served as negative control (non-differentiated cells). By the end of treatment periods, culture supernatants were collected and analyzed for the presence of helical peptide 620-633 released from the α1 chain of type I collagen, the substrate of OsteoAssay^TM^ human bone plate, by using a MicroVue helical peptide EIA kit. The effect of AC-PACs on bone resorption mediated by differentiated osteoclasts was recorded as a decrease in the release of collagen helical peptide compared to untreated control cells.

### 3.7. Statistical analysis

Data were recorded as means ± SD of triplicate samples. The statistical comparisons were performed using Student’s t-test with Bonferroni correction. The level of significance was set at p < 0.05. 

## 4. Conclusions

Our results support that naturally occurring proanthocyanidins, such as those from cranberry, have a valuable potential for therapeutic application in the treatment and prevention of bone loss related to inflammatory disorders as the periodontal disease. It seems that these plant-derived compounds would be able to interfere in osteoclastic cell maturation and physiology as well as in the bone matrix itself. Given their high biocompatibility, such substances may be easily introduced as a dietary supplement, which enables a large-scale application.
